# Supplementary material for: Food Frequency Questionnaire to Estimate Dietary Adherence in Hemodialysis Patients: A Pilot Study
Source: Nutrients. 2025 Oct 7;17(19):3161. doi: 10.3390/nu17193161 (PMC12526287; doi:10.3390/nu17193161)
Supplement: Supplementary file 1 [file nutrients-17-03161-s001.zip › nutrients-3895443-supplementary.pdf]

Supplementary Table S1. Frequency of food consumption among hemodialysis patients.

Food item	Never or <1/ Month	1–3/ month	1/ week	2–4/ week	5–6/ week	1/ day	2–3/ day	4–5/ day	>5/ day
Beef	12 (24)	24 (48)	12 (24)	1 (2)	0 (0)	0 (0)	1 (2)	0(0)	0 (0)
Pork	5 (10)	11 (22)	16 (32)	14 (28)	2 (4)	1 (2)	1(2)	0(0)	0 (0)
Chicken breast	4 (8)	11 (22)	16 (32)	14 (28)	5 (10)	0(0)	0 (0)	0 (0)	0 (0)
Turkey breast	11 (22)	19 (38)	14 (28)	3 (6)	3 (6)	0 (0)	0 (0)	0 (0)	0 (0)
Cod	20 (40)	19 (38)	7 (14)	4 (8)	0 (0)	0 (0)	0 (0)	0 (0)	0 (0)
Salmon	18 (36)	18 (36)	12 (24)	2 (4)	0 (0)	0 (0)	0 (0)	0 (0)	0 (0)
Smoked ham	9 (18)	9 (18)	8 (16)	11 (22)	10 (20)	1 (2)	2 (4)	0 (0)	0 (0)
Turkey ham	18 (36)	6 (12)	6 (12)	16 (32)	2 (4)	1 (2)	1 (2)	0 (0)	0 (0)
Sardines in oil	37 (74)	10 (20)	2 (4)	0 (0)	1 (2)	0 (0)	0 (0)	0 (0)	0 (0)
Herring in oil	26 (52)	18 (36)	4 (8)	2 (4)	0 (0)	0 (0)	0 (0)	0 (0)	0 (0)
Eggs	2 (4)	7 (14)	17 (34)	16 (32)	3 (6)	2 (4)	3 (6)	0 (0)	0 (0)
Natural yogurt	9 (18)	8 (16)	10 (20)	14 (28)	4 (8)	4 (8)	1 (2)	0 (0)	0 (0)
Milk 2%	9 (18)	8 (16)	7 (14)	8 (16)	6 (12)	8 (16)	4 (8)	0 (0)	0 (0)
Butter	5 (10)	7 (14)	8 (16)	6 (12)	4 (8)	10 (20)	7 (14)	2 (4)	1 (2)
Kefir	16 (32)	11 (22)	8 (16)	9 (18)	5 (10)	1 (2)	0 (0)	0 (0)	0 (0)
Processed cheese	11 (22)	11 (22)	12 (24)	13 (26)	1 (2)	2 (4)	0 (0)	0 (0)	0 (0)
Cream 12%	13 (26)	15 (30)	11 (22)	8 (16)	3 (6)	0 (0)	0 (0)	0 (0)	0 (0)
Goat cheese	39 (78)	9 (18)	2 (4)	0 (0)	0 (0)	0 (0)	0 (0)	0 (0)	0 (0)
Watermelon	31 (62)	11 (22)	4 (8)	3 (6)	1 (2)	0 (0)	0 (0)	0 (0)	0 (0)
Banana	24 (48)	11 (22)	8 (16)	4 (8)	2 (4)	0 (0)	1 (2)	0 (0)	0 (0)
Blueberries	15 (30)	21 (42)	10 (20)	4 (8)	0 (0)	0 (0)	0 (0)	0 (0)	0 (0)
Peach	23 (46)	20 (40)	2 (4)	4 (8)	0 (0)	1 (2)	0 (0)	0 (0)	0 (0)
Apple	6 (12)	7 (14)	5 (10)	11 (22)	5 (10)	14 (28)	2 (4)	0 (0)	0 (0)
Pear	8 (16)	20 (40)	10 (20)	6 (12)	4 (8)	2 (4)	0 (0)	0 (0)	0 (0)
Grapes	19 (38)	14 (28)	8 (16)	6 (12)	2 (4)	1 (2)	0 (0)	0 (0)	0 (0)
Tomato	11 (22)	9 (18)	10 (20)	9 (18)	6 (12)	2 (4)	3 (6)	0 (0)	0 (0)
Chicory	29 (58)	14 (28)	3 (6)	3 (6)	0 (0)	1 (2)	0 (0)	0 (0)	0 (0)
Cucumber	8 (16)	10 (20)	16 (32)	7 (14)	3 (6)	3 (6)	3 (6)	0 (0)	0 (0)
Potatoes	5 (10)	6 (12)	11 (22)	16 (32)	2 (4)	7 (14)	1 (2)	2 (4)	0 (0)
Zucchini	22 (44)	14 (28)	9 (18)	5 (10)	0 (0)	0 (0)	0 (0)	0 (0)	0 (0)
Carrot	5 (10)	13 (26)	12 (24)	14 (28)	5 (10)	1 (2)	0 (0)	0 (0)	0 (0)
Onion	6 (12)	9 (18)	9 (18)	20 (40)	5 (10)	1 (2)	0 (0)	0 (0)	0 (0)
White cabbage	13 (26)	19 (38)	11 (22)	4 (8)	3 (6)	0 (0)	0 (0)	0 (0)	0 (0)
Lentils	34 (68)	13 (26)	3 (6)	0 (0)	0 (0)	0 (0)	0 (0)	0 (0)	0 (0)
Green beans	24 (48)	19 (38)	6 (12)	1 (2)	0 (0)	0 (0)	0 (0)	0 (0)	0 (0)
Orange	20 (40)	7 (14)	10 (20)	9 (18)	3 (6)	1 (2)	0 (0)	0 (0)	0 (0)
Hazelnuts	21 (42)	22 (44)	5 (10)	2 (4)	0 (0)	0 (0)	0 (0)	0 (0)	0 (0)
Cauliflower	15 (30)	23 (46)	9 (18)	3 (6)	0 (0)	0 (0)	0 (0)	0 (0)	0 (0)
Bread roll	2 (4)	3 (6)	1 (2)	15 (30)	7 (14)	2 (24)	9 (18)	1 (2)	0(0)
Wheat bread	4 (8)	2 (4)	6 (12)	10 (2)	7 (14)	9 (18)	12 (24)	0 (0)	0 (0)
Pasta	0 (0)	10 (20)	18 (36)	14 (28)	6 (12)	1 (2)	1 (2)	0 (0)	0 (0)
Millet groats	27 (54)	11 (22)	5 (10)	7 (14)	0 (0)	0 (0)	0 (0)	0 (0)	0 (0)
Buckwheat groats	18 (36)	18 (36)	7 (14)	5 (10)	2 (4)	0 (0)	0 (0)	0 (0)	0 (0)
White rice	6 (12)	17 (34)	11 (22)	14 (28)	1 (2)	0 (0)	1 (2)	0 (0)	0 (0)
Whole-grain bread	18 (36)	13 (26)	9 (18)	4 (8)	2 (4)	2 (4)	2 (4)	0 (0)	0 (0)
Dark chocolate	23 (46)	16 (32)	6 (12)	3 (6)	1 (2)	1 (2)	0 (0)	0 (0)	0 (0)
Milk chocolate	22 (44)	11 (2)	13 (26)	4 (8)	0 (0)	0 (0)	0 (0)	0 (0)	0 (0)
Biscuits	10 (20)	19 (38)	15 (30)	6 (12)	0 (0)	0 (0)	0 (0)	0 (0)	0 (0)
Gummies	30 (60)	12 (24)	7 (14)	1 (2)	0 (0)	0 (0)	0 (0)	0 (0)	0 (0)
Hard candies	22 (44)	15 (30)	7 (14)	2 (4)	1 (2)	2 (4)	0 (0)	1 (2)	0 (0)
Sugary soft drinks	22 (44)	13 (26)	11 (22)	2 (4)	1 (2)	1 (0)	0 (0)	0 (0)	0 (0)
Energy drinks.	35 (70)	9 (18)	3 (6)	2 (4)	1 (2)	0 (0)	0 (0)	0 (0)	0 (0)
Black coffee	10 (20)	1(4)	1 (2)	4 (8)	3 (6)	19 (38)	9 (18)	1 (2)	1 (2)
Orange juice	17 (34)	10 (20)	14 (28)	3 (6)	3 (6)	2 (4)	1 (2)	0 (0)	0 (0)
Alcohol	32 (64)	14 (28)	4 (8)	0 (0)	0 (0)	0 (0)	0 (0)	0 (0)	0 (0)
